# ATR-FTIR spectroscopy as adjunct method to the microscopic examination of hematoxylin and eosin-stained tissues in diagnosing lung cancer

**DOI:** 10.1371/journal.pone.0233626

**Published:** 2020-05-29

**Authors:** Ruth Bangaoil, Abegail Santillan, Lara Mae Angeles, Lorenzo Abanilla, Antonio Lim, Ma. Cristina Ramos, Allan Fellizar, Leonardo Guevarra, Pia Marie Albano

**Affiliations:** 1 The Graduate School, University of Santo Tomas, Manila, Philippines; 2 Research Center for the Natural and Applied Sciences, University of Santo Tomas, Manila, Philippines; 3 University of Santo Tomas Hospital, Manila, Philippines; 4 Faculty of Medicine and Surgery, University of Santo Tomas, Manila, Philippines; 5 Divine Word Hospital, Tacloban City, Northern Leyte, Philippines; 6 Mariano Marcos Memorial Hospital and Medical Center, Ilocos Norte, Philippines; 7 College of Science, University of Santo Tomas, Manila, Philippines; 8 Faculty of Pharmacy, University of Santo Tomas, Manila, Philippines; Bar Ilan University, ISRAEL

## Abstract

Lung cancer remains the leading cause of cancer-related death worldwide. Since prognosis and treatment outcomes rely on fast and accurate diagnosis, there is a need for more cost-effective, sensitive, and specific method for lung cancer detection. Thus, this study aimed to determine the ability of ATR-FTIR in discriminating malignant from benign lung tissues and evaluate its concordance with H&E staining. Three (3) 5μm-thick sections were cut from formalin fixed paraffin embedded (FFPE) cell or tissue blocks from patients with lung lesions. The outer sections were H&E-stained and sent to two (2) pathologists to confirm the histopathologic diagnosis. The inner section was deparaffinized by standard xylene method and then subjected to ATR-FTIR analysis. Distinct spectral profiles that distinguished (*p*<0.05) one sample from another, called the “fingerprint region”, were observed in five (5) peak patterns representing the amides, lipids, and nucleic acids. Principal component analysis and hierarchical cluster analysis evidently clustered the benign from malignant tissues. ATR-FTIR showed 97.73% sensitivity, 92.45% specificity, 94.85% accuracy, 91.49% positive predictive value and 98.00% negative predictive value in discriminating benign from malignant lung tissue. Further, strong agreement was observed between histopathologic readings and ATR-FTIR analysis. This study shows the potential of ATR-FTIR spectroscopy as a potential adjunct method to the gold standard, the microscopic examination of hematoxylin and eosin (H&E)-stained tissues, in diagnosing lung cancer.

## Introduction

Lung cancer remains a global burden being the leading cause of cancer-related death in both men and women, accounting for 18.6% of total cancer-related death worldwide [1–3]. Since no effective screening method is available, the disease is usually diagnosed at an advanced stage, resulting to a poor 5-year survival rate estimated at 16% [4].

Lung cancer is a heterogenous group of tumors which can be divided into two (2) main categories: (1) the non-small cell lung carcinoma (NSCLC) which accounts for 85% of lung cancers and (2) the small cell lung carcinoma (SCLC) [5]. NSCLC is further subclassified into adenocarcinoma (40%), squamous cell carcinoma (30%), and large cell carcinoma (10%) based on their histogenetic and immunohistochemical characteristics [5]. The disease is caused by genetic and environmental factors, with smoking as the most consistent causative factor [6, 7]. Patients suspected of lung cancer usually experience persistent cough, dyspnea, shortness of breath, hemoptysis, chest pain, weight loss, and cachexia [8].

At present, a combination of radiological imaging such as chest x-ray, magnetic resonance imaging (MRI), low-dose computed tomography (LDCT), bronchoscopy, and histopathological examinations are conducted to diagnose lung cancer [9]. However, computed tomography (CT) screening is prone to false positive diagnosis, exposes healthy individuals to radiation, and its cost effectiveness is still uncertain [10]. MRI is currently the only technique that enables non-invasive whole body assessment without ionizing radiation but it is more susceptible to cardiac and respiratory motion artifacts [9]. Microscopic examination of hematoxylin and eosin (H&E)-stained biopsy specimen is currently the gold standard in diagnosing lung cancer [11]. However, sample preparation and slide processing are time-consuming, resulting in a slower turnover of results. In addition, diagnostic disagreement among pathologists have been noted, which may significantly influence prognosis, thus, indirectly affecting clinical interventions [12–16]. In the event that the diagnosis is not specific, pathologists would suggest immunohistochemical (IHC) staining of formalin fixed paraffin embedded (FFPE) tissue, within which a combination of positive and negative stains can aid in determining the specific diagnosis [17], thereby causing further delay in patient management.

The onset and progression of cancer cells are manifested at the molecular level which cannot be detected initially by conventional techniques until morphologic changes become evident [18]. Hence, there is an urgent need to develop a more sensitive and specific method to support the standard histopathological procedures to which patient treatment and management are dependent. One of the methods being considered at present is Fourier transform infrared (FTIR) spectroscopy given its sensitive and efficient structure elucidation capacity, leading to rapid and reliable turnover of result [19–21].

FTIR spectroscopy is based on the principle that when a sample is probed with an infrared (IR) beam, the functional groups within that sample will absorb the IR radiation, and the vibrational characteristics of each functional group will then be reflected [22]. The differences in the macromolecular composition of cells and tissues produce unique vibrational spectroscopic patterns (approximately 1800 cm^-1^–850 cm^-1^) which resemble the “fingerprint”, hence called the “fingerprint region”. This serves as basis for IR spectroscopic-based detection of human diseases [23].

There are three (3) FTIR sampling modes: transmission, transflection, and attenuated total reflection (ATR) [24]. In transmission mode, the IR radiation is transmitted through sample-substrate using IR-transparent materials such as BaF_2_ or CaF_2_ slides [23, 25], while in transflection mode, the IR radiation interacts with the sample and then back-reflected by the substrate using an IR-reflecting material like low-emissivity slides [25]. Lastly, in ATR mode, measurements are obtained from changes that occur when the reflected IR beam interacts with a sample placed on a higher refractive index crystal such as diamond or zinc selenide (ZnSe). The ATR mode is more advantageous over other FTIR sampling modes when analyzing biological samples because of its higher signal-to-noise ratio and reduced scattering [23, 25]. It also requires minimal or no sample preparation since the penetration depth of IR light is independent of sample thickness [26]. ATR mode can even be used for aqueous samples (biofluids) with minimal or reduced preparation compared to other FTIR sampling modes [23].

Over the past years, FTIR has been used to study different types of malignancy because of its ability to differentiate cancerous from healthy cells [23]. This technique is non-destructive to cells and is able to directly detect biochemical changes as compared to histopathological and immunohistochemical techniques which make use of stains or contrast agents for imaging [27]. The capacity of FTIR to discriminate colon cancer from healthy cells has been conducted previously [28, 29]. Grading prostate cancer by FTIR in conjunction with a principal component-discriminant function analysis (PC-DFA) algorithm has also been evaluated [30]. Classification of normal brain tissue from gliomas by FTIR has also been conducted [31]. FTIR has been tested for its potential as a rapid but accurate diagnostic method for differentiating malignant from benign thyroid nodules intra-operatively [18]. Further, its possible application in the early detection of lung cancer using sputum specimens has been demonstrated [32]. Thus, this study evaluated the potential of attenuated total reflectance-Fourier transform infrared (ATR-FTIR) spectroscopy as adjunct method to the gold standard, the microscopic examination of H&E-stained tissues, in the diagnosis of lung cancer.

## Materials and methods

### Ethical consideration

The researchers confirm that this study was specifically approved by the Institutional Review Board (IRB) of the two study sites–University of Santo Tomas Hospital, Manila (IRB2017-09-191-IS) and Mariano Marcos Memorial Hospital and Medical Center, Ilocos Norte (MMMH-RERC-15-006). Written informed consent from patients or their legal guardians has been waived by the IRB of USTH and MMMH-MC since the study was restricted to the use of archived FFPE specimens and did not involve invasive procedures nor pose risk or harm to subjects. The authors confirm that all methods were carried out in accordance with relevant guidelines and regulations.

### Sample preparation

FFPE cell or tissue blocks from patients with histologically confirmed lung cancer (*n* = 54) diagnosed from 2015 to 2017 at MMMH-MC and USTH were classified as malignant samples. Meanwhile, lung tissue samples histologically confirmed negative for malignant cells *(n =* 66*)* were classified as benign samples. The FFPE tissue blocks were cut into 5-μm thick sections using a microtome (Leica Biosystems, Germany), with three (3) adjacent sections mounted on a glass slide. The two outer sections were subjected to H&E staining and then sent to two (2) external evaluators (pathologists) blinded of the original diagnosis for validation. The pathologists were instructed to identify the H&E-stained tissue sections as either “malignant” or “benign” based on microscopic analysis and to mark the area where the malignant cells were located. The middle section was deparaffinized with xylol following standard protocols [33, 34], rinsed with water, and then left to dry overnight prior to spectral acquisition [35].

### Spectral measurement

Performance qualification (PQ) was done before any test was performed to ensure the clarity and accuracy of the generated infrared (IR) spectra. IR spectra were obtained using Bruker Alpha II Fourier transform IR (FTIR) spectrometer equipped with a platinum (ATR) single reflection diamond sampling module (Bruker Optics, Germany). Background spectrum was collected prior to scanning a new sample to improve signal-to-noise ratio. Deparaffinized tissue sections were pressed directly in contact with the diamond crystal’s surface (2 mm × 2 mm) using a single reflection snap ATR and an average of 48 scans were collected with 4 cm^-1^ spectral resolution. It must be noted that approximately 75% of lung cancer cases are diagnosed with advanced stage of the disease (stage III/IV) [36], which is associated with tumor size greater than 4.0 cm [37]. In this study, majority of the H&E-stained malignant sections were entirely cancer cells. Hence, the corresponding deparaffinized unstained tissue section was scanned fully to acquire spectral data. However, for H&E-stained slides marked by the pathologists on areas where cancer cells were concentrated, the corresponding deparaffinized unstained tissue section was only scanned along the indicated area. For the benign samples, at least 50% of the tissue section was scanned at random spots to acquire spectral data. Tissue sections were examined in the mid-infrared region (4000 cm^-1^–600 cm^-1^) and spectral profiles of the fingerprint region (1800 cm^-1^–850 cm^-1^) were further processed for multivariate analyses. Each sample was scanned three times to guarantee reproducibility.

### Data processing and analysis

Data were processed using OPUS 8.0 (Bruker Optics, Germany). All generated spectra were internally baseline corrected following rubber band method with 64 baseline points [38]. The median infrared spectra of both malignant and benign samples in the fingerprint region were calculated. Normality test was done on the wavenumbers that showed the most distinct changes using Shapiro-Wilk test to decide whether parametric or non-parametric statistic test should be used. Since all data followed a non-normal distribution, Mann-Whitney *U* test was carried out to determine their statistical significance (*p*-value<0.05). ATR-FTIR’s performance in terms of sensitivity, specificity, accuracy, positive predictive value (PPV) and negative predictive value (NPV) were also assessed using the histopathologic diagnosis of the hospital study sites as standard. Using the infrared wavenumbers as markers for biochemical changes, we performed unsupervised multivariate analytical methods particularly principal component analysis (PCA) and hierarchical cluster analysis (HCA) as well as supervised linear discriminant analysis (LDA) to assess the diagnostic ability of ATR-FTIR in differentiating malignant from the benign sample groups. Moreover, Kendall coefficient of concordance was computed to assess the inter-rater agreement between pathologists and ATR-FTIR. All statistical analyses were performed using XLSTAT (Addinsoft, USA) except for the HCA dendrogram which was generated using PAST 3.24 software (Oslo, Norway).

## Results

### Samples

Only specimens in diagnostic concordance among external evaluators and original diagnosis of the study sites were considered for FTIR analysis. From the 120 retrieved FFPE samples, only 44 malignant and 53 benign FFPE cell or tissue sections met the inclusion criteria, therefore were the only ones subjected to FTIR analysis. The clinical characteristics of the samples were retrieved from medical records and histopathologic reports (**Table 1**). Majority of the malignant specimens had NSCLC (*n* = 32; 72.7%) as final diagnosis, with pleural and lung tissues as main biopsy source. The benign samples selected for this study were principally negative for malignant cells (*n* = 32; 60.4%) and were mainly from pleural tissues and effusions.

**Table 1:** Clinical characteristics of the samples.

Diagnosis*	Histopathologic classification	Specimen
**Malignant** (*n* = 44)	Non-small cell lung cancer (NSCLC)	Pleural effusion (*n* = 3)
** **	*Adenocarcinoma* (*n =* 20)	Pleural tissue (*n* = 17)
	*Squamous cell carcinoma* (*n* = 9)	Lung tissue (*n* = 14)
	*Large cell carcinoma* (*n* = 3)	Bronchial mass (*n* = 4)
	Small cell lung cancer (SCLC) (*n* = 3)	Chest wall mass (*n* = 2)
	Histopathologic classification not indicated	Thoracic biopsy (*n* = 2)
	in hospital records (*n =* 9)	Deep cervical node (*n* = 2)
	* *	
**Benign** (*n* = 53)	Negative for malignant cells (*n* = 32)	Pleural effusion (*n* = 32)
	Chronic granulomatous disease (*n* = 6)	Pleural tissue (*n* = 20)
	Tuberculous pleuritis (*n* = 9)	Thoracic fluid (*n* = 1)
	Chronic inflammation, fibrosis (*n* = 6)	

*Retrieved from histopathologic reports of hospitals. Diagnosis was based on the microscopic analysis of H&E-stained specimens, which is currently the gold standard. Only specimens in diagnostic concordance among external evaluators and the original diagnosis were considered for FTIR analysis.

### Spectral analysis of significant wavenumbers in the fingerprint region

**Fig 1:**
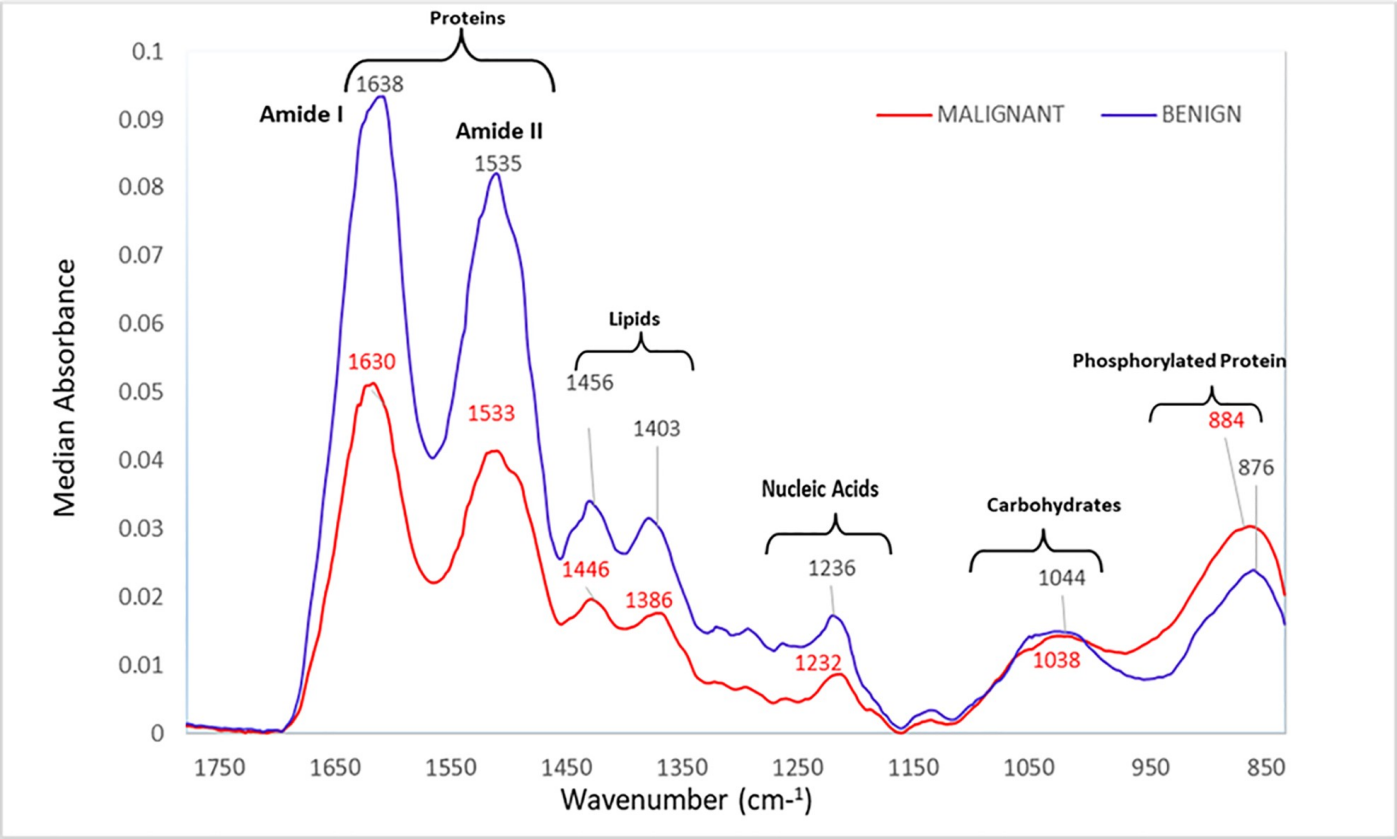
Median ATR-FTIR absorbance spectra of the malignant (*n* = 44) and benign (*n* = 53) lung samples in the fingerprint region showing distinct changes in the spectral absorption bands of proteins, lipids, and nucleic acids.

Median absorbance spectra of the 44 malignant and 53 benign samples in the fingerprint region is presented in **Fig 1**. A non-normal distribution of median absorbance spectra was observed between samples as analyzed from Mann-Whitney *U* and Shapiro-Wilk test. This test of homogeneity showed that the peak wavenumbers of the malignant cases were significantly different from benign samples particularly in the amides, lipids, and nucleic acid regions (*p*<0.0001).

It could be observed that median spectral absorbance of malignant samples were significantly reduced (*p*<0.0001) compared to benign samples at ~1638 cm^-1^/1630 cm^-1^, ~1535 cm^-1^/1533 cm^-1^, ~1456 cm^-1^/1446 cm^-1^, ~1403 cm^-1^/1386 cm^-1^, and 1236 cm^-1^/1232 cm^-1^. However, the spectral absorption bands of malignant and benign samples in ~1044 cm^-1^/1038 cm^-1^ and ~884 cm^-1^/876 cm^-1^ regions showed no significant differences.

Prominent peaks in the fingerprint region and their corresponding proposed vibrational modes and molecular sources are summarized in **Table 2** [39, 40]. Comparison of the peak positions and absorbance of the two groups (malignant versus benign) are presented in **Table 3.** The benign samples showed significantly higher absorbance peaks (*p*<0.0001) in the amide I, amide II, lipid, and nucleic acid regions compared to malignant samples. No statistically significant difference (*p*>0.05) was seen between the two groups in the carbohydrates and phosphorylated protein fingerprint regions.

**Table 2:** Frequency (cm^-1^) assignment, proposed vibrational mode and molecular source of prominent peaks observed within the fingerprint IR region.

Peak position		Proposed vibrational mode	Proposed molecular source*
**Benign**	**Malignant**	** **	** **
**1638**	1630	Amide I, C = O stretch	Proteins
**1535**	1533	Amide II, C-N stretch	Proteins
**1456**	1446	COO^-^ stretch, C-H bend	Lipids
**1403**	1386	COO^-^ stretch, C-H bend	Lipids
**1236**	1232	PO4^-^ stretch	Nucleic Acids
**1044**	1038	C-O stretch, C-O bend	Carbohydrates
876	**884**	PO4 ^=^ stretch, C-C stretch	Protein

*References [39, 40]; Values in bold refer to significantly higher peak absorbance.

**Table 3:** Comparison of the spectrum variables between malignant and benign lung samples in the fingerprint region (1800 cm^-1^ to 850 cm^-1^).

Peak position	Benign	Peak position	Malignant	*p-*value*
Mean abs ± SD		Mean abs ± SD		
1638 cm^-1^	0.097 ± 0.022	1630 cm^-1^	0.052 ± 0.013	**< 0.0001**
1535 cm^-1^	0.084 ± 0.019	1533 cm^-1^	0.044 ± 0.009	**< 0.0001**
1456 cm^-1^	0.034 ± 0.007	1446 cm^-1^	0.019 ± 0.004	**< 0.0001**
1403 cm^-1^	0.033 ± 0.007	1386 cm^-1^	0.016 ± 0.003	**< 0.0001**
1236 cm^-1^	0.018 ± 0.007	1232 cm^-1^	0.008 ± 0.004	**< 0.0001**
1044 cm^-1^	0.017 ± 0.010	1038 cm^-1^	0.018 ± 0.011	0.808
876 cm^-1^	0.026 ± 0.019	884 cm^-1^	0.035 ± 0.023	0.099

*Significant if *p-*value is less than 0.05 (*p*<0.05) using Mann-Whitney *U* test (two-tailed).

### Multivariate analysis of FTIR spectra

**Fig 2:**
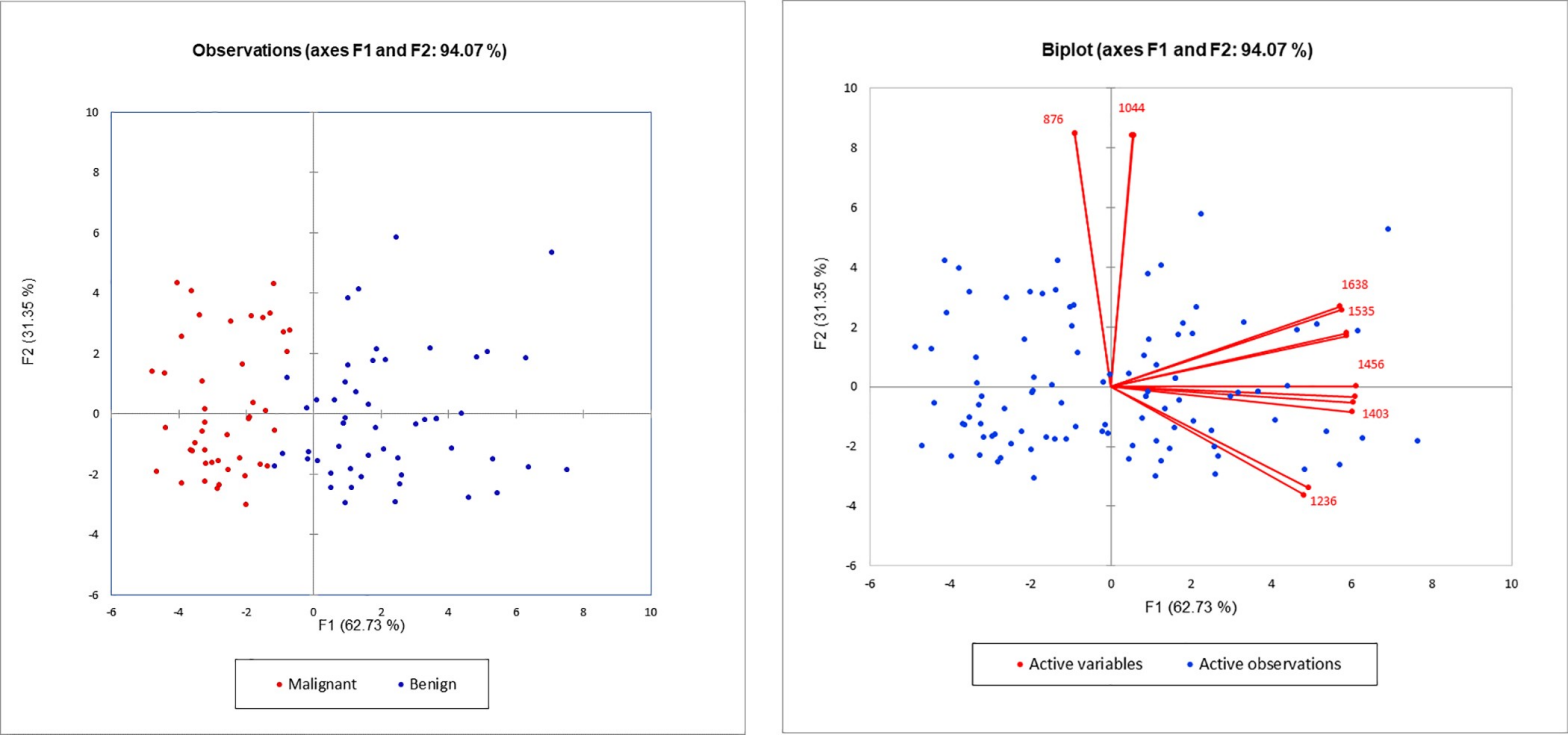
**A**. Principal component analysis (PCA) scatter plot of the significant wavenumbers in the fingerprint region showing distinct separation between malignant and benign samples at 99% CI. **B.** PCA biplot of the significant wavenumbers of malignant and benign samples in the fingerprint region showing PC scores and loadings of variables (wavenumbers, cm^-1^). F1 was mainly due to amides (1638 cm^-1^ and 1535 cm^-1^), lipids (1456 cm^-1^, 1403 cm^-1^), and nucleic acids (1236 cm^-1^) while F2 was mainly due to the carbohydrates (1044 cm^-1^) and phosphorylated proteins (876 cm^-1^).

PCA of the significant wavenumbers in the fingerprint region was done using XLSTAT (Addinsoft, USA). Two (2) principal components, F1 and F2, accounted for 94.07% (62.73%+31.35%) of the total variance in the data obtained from both malignant cases and benign controls (**Fig 2A)**. The PCA biplot (**Fig 2B)** shows that F1, which explained 62.73% of the variance, was mainly due to the amides (1638 cm^-1^, 1535 cm^-1^), lipids (1456 cm^-1^, 1403 cm^-1^), and nucleic acids (1236 cm^-1^). The positive loadings of 1638 cm^-1^ and 1535 cm^-1^ could correspond to the high levels of β-sheet secondary structures of proteins. F2, which explained 31.35% of the variance, was mainly attributed to the carbohydrates (1044 cm^-1^) and phosphorylated protein (876 cm^-1^). Results show distinct separation of the malignant from the benign samples at 99% confidence interval (CI).

**Fig 3:**
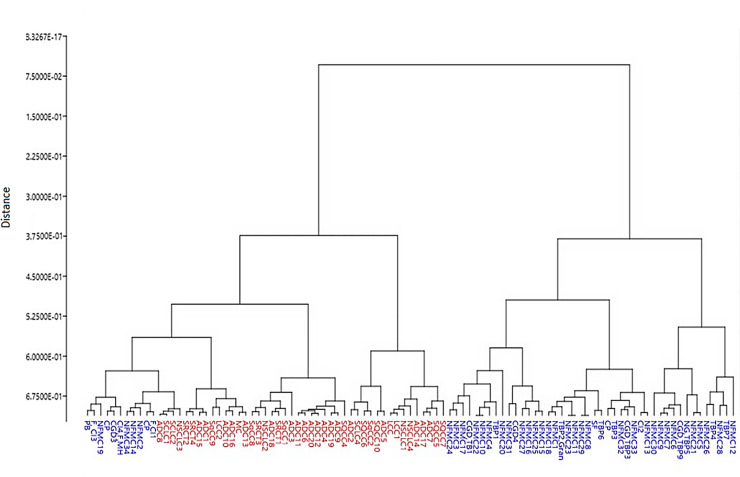
Hierarchical component analysis (HCA) dendrogram of malignant *(red)* and benign *(blue)* lung samples showing 2 major clusters. The first major cluster contains all the malignant samples (*n* = 44) with a sub-cluster of benign samples (*n* = 11) diagnosed with pulmonary bulla, chronic inflammation, fibrosis, chronic granulomatous disease, and pulmonary tuberculosis. The second cluster consists of the benign samples (*n* = 42).

The HCA dendrogram (**Fig 3)** was generated using Euclidean distance matrix and Ward’s method. HCA allows visualization of the overall grouping structure and consequently subgroups the spectra according to their similarities. The dendrogram shows two major clusters, suggesting that the wavenumbers identified in the fingerprint region can discriminate malignant from the benign samples with high accuracy. Although the HCA dendrogram demonstrates a clear separation between malignant and benign samples, few benign samples (*n* = 11) clustered with the malignant group. However, it must be noted that these 11 specimens were also diagnosed with other respiratory diseases such as chronic granulomatous disease, chronic inflammation, pulmonary bullae, fibrosis, or pulmonary tuberculosis.

### Linear discriminant analysis of significant wavenumbers in the fingerprint region

LDA was carried out to construct a predictive model for classifying samples into two groups–malignant and benign. This is based on the principle that LDA generates discriminate functions that result in maximal variance between groups but minimal variance within each group. Thus, the cluster plot generated could be considered as prototype diagnostic classifier [41].

A receiver operating characteristic (ROC) curve was created to assess the predictive ability of LDA. The obtained area under the curve (AUC) value was 0.9698, (99% CI, 0.92–1.00000), indicating that malignant cases were well-discriminated from the benign samples using this classification model. AUC values close to 1 indicate that the model is a very good classifier.

In this study, LDA results were concordant with histopathology-based diagnosis except for the four (4) samples diagnosed as benign by all pathologists but were classified as malignant by ATR-FTIR spectroscopy. Moreover, one (1) sample histologically classified as malignant was interpreted as benign by this technology.

The diagnostic performance of FTIR was also computed based on the outputs of the LDA. Sensitivity, specificity, and accuracy rate of ATR-FTIR in comparison with the gold standard–the microscopic analysis of H&E-stained biopsies–were calculated as 97.73%, 92.45%, and 94.85%, respectively. Moreover, ATR-FTIR showed a positive predictive value (PPV) of 91.49%, and negative predictive value (NPV) of 98.00%.

### Diagnostic concordance between pathologists and ATR-FTIR reading

Kendall coefficient of concordance (*W*), a non-parametric statistic tool, was used to measure agreement among pathologists and ATR-FTIR spectroscopy. *W* values equal to zero (0) and one (1) indicate no agreement and perfect agreement, respectively. Results of this study revealed strong agreement (*W* = 0.7194) among pathologists and ATR-FTIR spectroscopy, thus further demonstrating the potential of this technique in diagnosing malignancy of the lungs.

## Discussion

Lung cancer is a serious illness that is accountable for the highest portion of cancer-related death worldwide. Most cases are diagnosed at an advanced stage (stage III or IV) due to the late onset of signs and symptoms resulting in overall low survival of patients from only about 8–12 months [42]. Treatment options highly depend on a pathologist’s diagnosis, the molecular characteristics of the disease, as well as tumor stage and patient´s overall health status. In this sense, a rapid and accurate assessment of the disease is critical to determine the most appropriate treatment for lung cancer patients to reduce lung cancer mortality.

In the clinical setting, varied specimens which represent the human lung (pleural effusion, pleural tissue, lung tissue, bronchial mass, chest wall mass, thoracic biopsy, and deep cervical node) can be collected and submitted to the laboratory to screen for lung malignancy. For example, pleural effusions contain minute lung tissue fragments that accumulate in the pleural cavity. In such instances, a cell block is prepared by centrifugation of this specimen to recover the lung tissue fragments, formalin fixed, processed, and then paraffin-embedded following standard protocols. This formalin fixed paraffin embedded (FFPE) cell block is then sectioned using a microtome, stained with hematoxylin and eosin (H&E), and analyzed by a pathologist based on morphological characteristics. But with FTIR, there is no need for the staining step and analysis is based on biomolecular changes in cancer cells. It can also be observed that half of the specimens representing the benign group were pleural effusions since hospital protocol dictates that surgical resection is not required for benign diseases. Overall, the specimens selected for this study represent the human lung and have been confirmed as either benign or malignant by two external evaluators.

The ability of ATR-FTIR spectroscopy to directly probe the biochemical and structural composition of tissues without the use of stains or other contrast agents provides an ideal platform by which the macromolecular chemistry associated with disease can be investigated [43, 44]. In this study, ATR-FTIR spectroscopy was shown to successfully identify malignant lung cells or tissues by probing the biochemical changes occurring on the tissues during carcinogenesis.

The significant changes (*p*<0.0001) observed at ~1638 cm^-1^ and ~1535 cm^-1^ indicate alterations in the normal protein configuration of the lung tissue. Proteins play a key role in regulating cell metabolic activity, and for this reason, the changes observed in their spectral absorbance suggest disorders in the cell’s physiology in the case of malignancy [45].

Amide I is very sensitive, which is why it is used to study the secondary structure of proteins. Majority (80%) of the changes in the band position could have resulted from stretching vibrations of C = O group while a small impact could also be due to stretching vibrations of C-N group and bending vibrations of the N-H group in the β-sheet structure of proteins [39]. Peaks at ~1535 cm^-1^, which correspond to amide II, may be largely due to the coupling of CN stretching and in-plane bending of the N-H group [46]. The changes in the hydrogen bonding of the peptide group and consequently in the molecular geometry of the proteins may instigate cell damages in the protein folding, resulting in a definitive loss of protein biological functions or mutations [24].

Increase in absorption at ~884 cm^-1^ due to alterations in phosphorylated proteins suggest that more extensive phosphorylation occurs in malignant compared to benign samples. Protein phosphorylation is particularly important in cellular processes such as protein synthesis, cell division, signal transduction, cell growth, development and apoptosis. Alterations or modifications in the phosphorylation process can lead to chronic inactivation of the protein itself, which in turn can transform healthy cells into cancer cells [47]. Increase in phosphorylated protein in the biological fingerprint spectrum have also been presented previously [32, 46].

Broad region of peaks observed in the absorption bands at ~1456 cm^-1^ to ~1403 cm^-1^ regions are associated with vibrations of methyl and methylene groups in fatty acids and cellular membranes [48]. Decreased absorption bands of malignant samples could be due to their rapid growth that needed to consume large amounts of fats and disorderly utilized exogenous lipids as nutrients, resulting in reduced C-H and C-O stretching vibrations [28]. Alterations in the lipid content of malignant cells affect cell membrane fluidity, thus the increased survival as well as migration, invasion and tumor angiogenesis of the malignant cells [49].

Similarly, the peak intensity change at ~1236 cm^-1^ is due to PO_2_ asymmetric stretching of nucleic acids [50]. The decrease in the absorption bands in the nucleic acid region may be related to necrosis and apoptosis of malignant cells or DNA released by them [38].

The peak intensity change at ~1044 cm^-1^ may be due to C–O stretching coupled with bending in carbohydrates [51, 52]. During carcinogenesis, it is assumed that glycogen levels decrease, thus the reduction in the intensity of spectral absorption [53]. Studies suggest that glycogen level in malignant cells can be considered as good differentiation marker against benign cells [51, 54]. Increasing pieces of evidence indicate that in a tumor microenvironment, malignant cells accumulate glycogen as stored energy to enable survival during adverse conditions such as prolonged hypoxia and glucose deprivation as well as to sustain metastases [55]. In the current study, peak intensity in the absorbance regions associated with carbohydrates was not significantly different between the malignant and benign samples. Probably, the malignant samples had used up the stored glycogen for survival or even to sustain metastases. It could also be due to the different subclasses of lung carcinoma samples that were analyzed as well as the variation in the proportion of different cells present in each tissue section [54]. It has been reported that glycogen levels differ depending on the subclass, degree, and grading of lung carcinoma [51]. It is worthy to note at this point that the ATR detector attached to the FTIR spectroscopy used in this study was independent of sample thickness [56]. Thus, the intensity of the spectral absorption of the sample was proportional to the contents of the tissue being examined. However, given the characteristic heterogeneity of lung cancer [57], the exact sources (whether the carcinoma cells, extracellular matrix, or mesenchymal cells such as fibroblasts, infiltrating immune cells and vasculature [58]) of the differences in IR spectra between malignant and benign tissues cannot be fully elucidated. Further molecular and biochemical analyses should be designed to answer the aforementioned issues.

PCA successfully segregated the malignant from benign samples based on the loadings and factor scores of the significant wavenumbers in the fingerprint region. HCA clustered the samples into two (2) distinct clusters of malignant and benign groups according to their level of similarities. Although there was a distinct separation of the two (2) groups, a small group of benign samples (*n* = 11) subclustered with the malignant samples. Clinicopathologic records of these samples revealed pulmonary tuberculosis (PTB) and chronic granulomatous disease (CGD). Interestingly, lung cancer has been found to also arise from chronic inflammation that has led to metaplasia of epithelium in the cavities, in the calcified lymph nodes, and in old scars in the bronchi as consequences of pulmonary tuberculosis (PTB), chronic granulomatous disease (CGD), and other inflammatory lung diseases [54]. One study [55] presented experimental evidence that chronic *Mycobacterium tuberculosis* infection could initiate the development of lung carcinoma.

Since the initial presentation of lung cancer often mimics PTB or CGD [56], the characteristics of lung cancer are often masked [57], thereby affecting the diagnosis and delaying the treatment. This has become common among developing countries like the Philippines due to lack of awareness, inadequate infrastructure, and socio-economic factors [58]. Since the ethics permit for this study was restricted to the analysis of retrospective samples, any information on what transpired to the patients with diagnosis of a benign lung disease was not made available. Hence, a longitudinal study can be planned to follow up on this group of patients. And given the results of the current study and what other studies have observed, it can be assumed that ATR-FTIR can possibly detect lung malignancy camouflaged by another lung disease.

With LDA, four (4) of the samples diagnosed by the pathologists as benign were classified by FTIR as malignant. Interestingly, these are the same samples that sub-clustered with the malignant group in the HCA dendrogram. As mentioned earlier, they were also diagnosed with PTB. In case their lung cancer was masked by the infection, then the findings of this study support the idea that biochemical changes precede morphological changes within cells and tissues [59]. Hence, the ATR-FTIR was able to detect these carcinogenesis-associated biomolecules before morphological changes in tissues become detectable.

Aside from tissue samples, studies have also demonstrated that plasma can be a less invasive approach for molecular tumor profiling without having to obtain tumor tissue [60–62]. But for economically less-developed countries, these molecular techniques may not be affordable for clinical use. Hence, FTIR can be a cheaper alternative for detecting these molecular biomarkers based on their unique vibrational patterns.

The advantages offered by ATR-FTIR spectroscopy in the clinical setting would be the relative low-cost of instrumentation since this technique is non-analyst dependent. Being non- destructive in nature, the samples could be reused for further analysis. Compared to the gold standard, FTIR imaging does not make use of dyes or labelling methods to visualize the components in the sample [26]. It can even speed up the diagnostic process through the application of computer-based technologies.

While the current study shows the potential of ATR-FTIR as adjunct method to the gold standard when pathologists cannot arrive to a concordant diagnosis, robust clinical studies would still be required to bring this technology into the clinical setting. For instance, due to inadequate resources, this study was conducted using only the basic type of FTIR routine spectrometer, which is not attached to a microscope and with limited spatial resolution. To advance the present findings, it is recommended that additional batch of new samples are to be tested with an infrared microscope in order to detect vibrational motions of molecules within very restricted regions, *i*.*e*., the suspicious cells [26].

In the current study, the analysts relied on the pathologists’ specifications, hence the entire area identified to contain cancer cells (around 700 to a thousand cells) was scanned to obtain spectral data, possibly including the tumor microenvironments and nearby morphologically normal cells. Yet, it can be argued that contamination by non-neoplastic cells was less likely for FFPE cell blocks [63]. Moreover, the ATR-FTIR could have been useful in detecting carcinogenesis-associated biochemical changes in morphologically normal-looking cells and their microenvironments [64].

In summary, results of the current study show that the significant wavenumbers identified in the fingerprint region coupled with chemometric analysis can effectively discriminate between malignant and benign lung samples. The unique infrared spectrum of absorption in the amide, lipid, and nucleic acid regions discriminated the malignant from benign samples. Hence, ATR-FTIR spectroscopy can be a potential adjunct method to the gold standard in the diagnosis of lung cancer.
